# Autosomal recessive polycystic kidney disease diagnosed in fetus

**DOI:** 10.4103/0970-1591.33738

**Published:** 2007

**Authors:** Joseph Thomas, A. P. Manjunath, Lavanya Rai, Ranjini Kudva

**Affiliations:** Department of Urology, Kasturba Medical College, Manipal, India; * Department of OBGYN, Kasturba Medical College, Manipal, India; ** Department of Pathology, Kasturba Medical College, Manipal, India

The presence of isolated large and hyperechoic fetal kidneys suggest polycystic kidney disease. The antenatal diagnosis has to be made without doubt as it has serious implications in the continuation of pregnancy, evaluation of family members and genetic counseling for the family. We present the features of autosomal recessive polycystic kidney disease (ARPKD) diagnosed antenatally by ultrasound and confirmed by fetal autopsy.

## CASE REPORT

**Figure 1:**
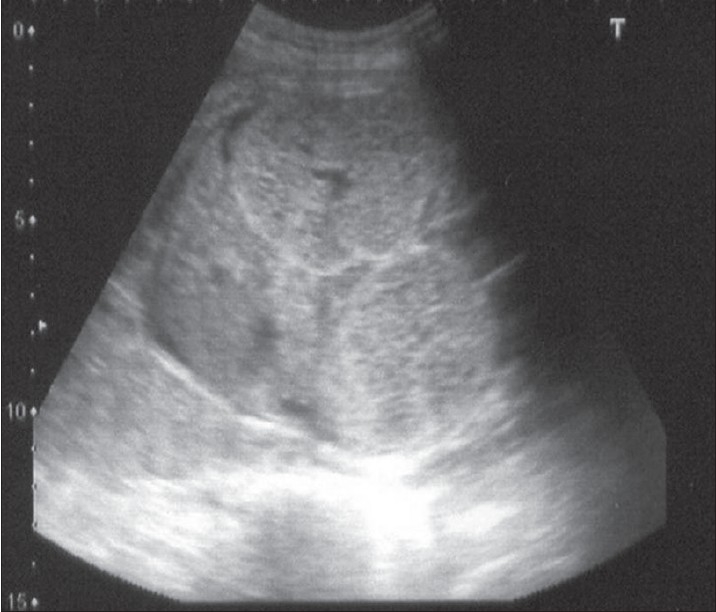
Antenatal ultrasound at 24 weeks showing bilateral, symmetrically enlarged, echogenic kidneys filling the fetal abdomen. Liver is normal

**Figure 2:**
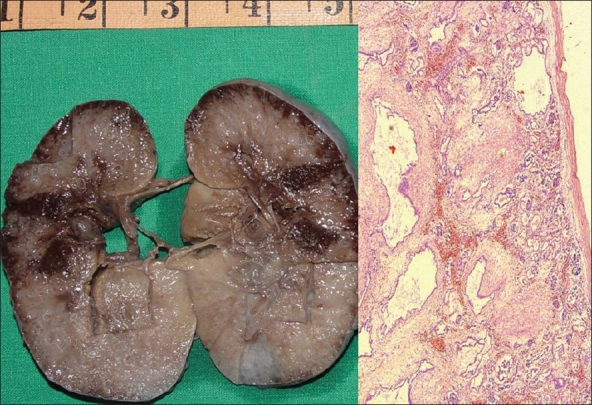
A) Gross specimen of right kidney measuring 10 × 7 × 4 cms. The cut surface is spongy with poor corticomedullary differentiation. There are multiple, tiny cysts with some at right angles to the cortical surface. B) Photomicrograph showing numerous cysts lined by a single layer of low cuboidal epithelial cells with thick peritubular mesenchyme. The glomeruli are normal (H and E, ×40)

A 27-year-old primi gravida who had normal pregnancy till 24 weeks of gestation was referred for fetal renal evaluation. The fetal scan is shown in Figure 1. There were bilateral, symmetrically enlarged, echogenic kidneys filling the fetal abdomen. The urinary bladder was not visible and the amniotic fluid index was 9. There were no other anomalies noted and fetal liver was normal. Both the parents were normal on evaluation and there was no family history of renal diseases on the maternal or paternal side. The patient and the family were counseled about the possibility of autosomal recessive polycystic kidney disease. As the amniotic fluid was normal, the patient was advised to undergo a repeat evaluation. Repeat scanning at 28 weeks showed that the kidneys had become more hyperechogenic with evidence of severe oligohydramnios and fetal ascites. The patient went into premature contractions and delivered vaginally at 29 weeks. The delivery was difficult due to the large size of the fetal kidneys. The fetus showed features of Potter's facies. Fetal autopsy was done. The gross and microscopic appearance of the right kidney is shown in Figure 2. Both the kidneys were enlarged, each measuring 10 × 7 × 4 cm. The cut surface showed spongy appearance with poor corticomedullary differentiation. There were multiple tiny cysts with some at right angles to the cortical surface. Microscopy of both kidneys showed numerous cysts lined by a single layer of low cuboidal epithelial cells with thick peritubular mesenchyme. The glomeruli appeared normal. Liver showed normal histology. The gross and microscopic features were in favor of ARPKD.

## DISCUSSION

The hyperechogenicity of the kidneys can be diagnosed after 17 weeks gestation and results from the presence of multiple micro cysts, dysplasia or tubular dilatation.[1] The differential diagnosis should take into account family history and the presence of associated anomalies. If there are no other malformations in the fetus the main diagnosis is polycystic kidney disease - recessive or dominant.[1] In dominant polycystic kidney disease the fetus will show macro cysts and the amniotic fluid is normal.

ARPKD is the most common heritable cystic renal disease occurring in infancy, with mutations of a single localized gene in an area in Chromosome 6 (PHKD1).[2] PHKD1 gene is expressed at high levels in fetal and adult kidneys and at lower levels in the liver and this corresponds to the principal sites of the disease. The characteristic pathologic changes occur in the kidneys and the liver with a reciprocal relationship between the degree of renal and hepatic involvement. The hallmark manifestation in the liver is congenital hepatic fibrosis with varying degrees of biliary ectasia and periportal fibrosis. Kidneys show subcapsular cysts, representing ectasia of the collecting tubules. In the cross-section, these dilated tubules can be seen in a radial arrangement extending from the calyx to the capsule. There is epithelial hyperplasia along the collecting ducts and these hyperplastic cells undergo a functional change from resorption to secretion. The combination of epithelial hyperplasia and fluid secretion results in ductal ectasia. Depending on the extent of ductal involvement there is a wide variability of renal dysfunction.

Molecular prenatal diagnostic techniques can be used to detect ARPKD in early pregnancy.[3] Serial ultrasound evaluation starting at 15 weeks can be used as a screening modality. The characteristic findings may not be apparent until late second trimester as in this case. There is a spectrum of findings in ARPKD which are better depicted using high-resolution ultrasound techniques.[4] The characteristic findings in ultrasound are enlarged, homogenously hyperechogenic kidneys with the absence of corticomedullary differentiation and difficulty in identifying fetal bladder.[5] The liver is usually normal in echogenicity. The increased echogenicity in the kidneys is due to the return of the sound waves from the enormous number of interfaces created by tightly compacted collecting ducts. These cases have significant nephromegaly with about 90% of the ducts involved, which may impede delivery. The impairment in renal function leads to oligohydramnios leading to pulmonary hypoplasia, club foot and Potters’ facies. Repeat sonographic measurement of the length of the kidneys appears to be a useful parameter to diagnose ARPKD.[4] The prognosis of these antenatally detected cases is bleak with death occurring within the first two months due to uremia or respiratory failure.

## References

[1] Chaumoitre K, Brun M, Cassart M, Maugey-Laulom, Eurin D, Didier F (2006). Differential diagnosis of fetal hyperechogenic cystic kidneys unrelated to renal tract anomalies: A multicentre study. Ultrasound Obstet Gynecol.

[2] Zerres K, Mucher G, Becker J, Steinkamm C, Rudnik-Schoneborn S, Heikkila P (1998). Prenatal diagnosis of autosomal recessive polycystic kidney disease (ARPKD): Molecular genetics, clinical experience and fetal morphology. Am J Med Genet.

[3] Bergmann C, Senderek J, Schneider F, Dornia C, Kupper F, Eggermann T (2004). PKHD1 mutations in families requesting prenatal diagnosis for autosomal recessive polycystic kidney disease (ARPKD). Hum Mutat.

[4] Traubici J, Daneman A (2005). High-resolution renal sonography in children with autosomal recessive polycystic kidney disease. AJR Am J Roentgenol.

[5] Roume J, Ville Y (2004). Prenatal diagnosis of genetic renal diseases: Breaking the code. Ultrasound Obstet Gynecol.

